# A new minute *Pristimantis* (Amphibia: Anura: Strabomantidae) from the Andes of southern Ecuador

**DOI:** 10.1371/journal.pone.0202332

**Published:** 2018-08-29

**Authors:** Paul Székely, Juan Sebastián Eguiguren, Diana Székely, Leonardo Ordóñez-Delgado, Diego Armijos-Ojeda, María Lorena Riofrío-Guamán, Dan Cogălniceanu

**Affiliations:** 1 Universidad Técnica Particular de Loja, Departamento de Ciencias Biológicas, EcoSs Lab, San Cayetano Alto, calle Marcelino Champagnat s/n, Loja, Ecuador; 2 Association Chelonia Romania, Bucharest, Romania; 3 Faculty of Natural and Agricultural Sciences, Ovidius University Constanța, Constanța, Romania; 4 Laboratory of Fish and Amphibian Ethology, Behavioural Biology Group, Freshwater and OCeanic Science Unit of ReSearch (FOCUS), University of Liège, Liège, Belgium; Universitat Trier, GERMANY

## Abstract

We describe a new rainfrog species (*Pristimantis*), from the wetland complex Oña, Nabón, Saraguro and Yacuambi, in the Andes of southern Ecuador, at altitudes ranging between 3000–3400 m a.s.l. *Pristimantis tiktik* sp. nov. is a small frog, displaying sexual dimorphism (the males with dorsum of various shades of gray, brown, orange or green and a whitish or pinkish yellow venter; females with brownish gray or gray dorsum and a reticulated white and black venter), with SVL ranging between 19.7–20.4 mm in females (n = 3) and 16.1–18.4 mm in males (n = 6). The skin on dorsum is tuberculated, that on venter is coarsely areolate, dorsolateral folds are absent, tympanic membrane is absent but the tympanic annulus is evident, cranial crests are absent, discs on fingers just slightly expanded, heel is lacking enlarged tubercles, inner edge of tarsus is bearing a long fold, Toe V is slightly longer than Toe III and the iris coloration is bronze with fine black reticulations. The males have a large subgular vocal sac that extends onto the chest and vocal slits but lack nuptial pads. The unique advertisement call consists of long duration series of periodically repeated clicks: “tik”. Molecular analyses place the new species in the recently resurrected *P*. *orestes* group, as the sister species of the assemblage that contains *P*. *bambu*, *P*. *mazar*, *P*. *simonbolivari* and an undescribed species.

## Introduction

The past decades triggered an explosion of new data on species distribution and detailed maps of climatic and environmental variables, in addition to significant advances in the reconstruction of the tree of life [1]. There is a striking difference between temperate and tropical species diversity [2], and half of the currently recognized amphibian species have been described only after 1960 [3]. The rate of description varied; for example, until 1960, the Neotropics appeared to have an overall diversity similar to Asia or North America, but since then the number of recognized species here has tripled [3].

*Pristimantis* Jiménez de la Espada, 1870, currently contains 526 described species [4] being the largest genus among all vertebrates [5]. Its remarkable diversity could probably be explained by the evolution of direct development, allowing individuals not to depend on water bodies for reproduction, and thus making them fit for niches unoccupied by other amphibians [6]. Additionally, low dispersal abilities and high sensitivity to climatic factors, such as humidity and temperature, have favored allopatric speciation [7]. Another important feature of the genus is its highly variable body size, varying from 13.7 mm to 73.0 mm, a factor also likely to have promoted the exploitation of various niches [8].

*Pristimantis* is the result of a mega-radiation event [9] and contains 6.7% of the known amphibian species and 7.6% of all anuran species. In Ecuador, there are 227 *Pristimantis* species currently registered, which represent 38.2% of the known amphibian species and 40.5% of all anuran species of the country [10]. The diversity of this genus is far from reaching a plateau, as the description rate has not slowed down, especially since the advent of molecular techniques has facilitated the discovery of cryptic diversity (e.g. [11–13]). Herein we describe a new tiny species of *Pristimantis* from the wetland complex of Oña, Nabón, Saraguro and Yacuambi, located in the Andes of southern Ecuador. We assign this new species to the recently redefined *Pristimantis orestes* group [14].

## Materials and methods

### Ethics statement

This study was carried out in strict accordance with the guidelines for use of live amphibians and reptiles in field research compiled by the American Society of Ichthyologists and Herpetologists, The Herpetologists' League and the Society for the Study of Amphibians and Reptiles. Research permit was issued by Ministerio del Ambiente del Ecuador (MAE-DNB-CM-2015-0016). This study was evaluated and approved by the Ethics Committee of Universidad Técnica Particular de Loja (UTPL-CBEA-2016-001).

### Study site and specimen collection

Fieldwork was carried out in 2016 (July 7, 8 and 23, August 2, 4 and 10), 2017 (May 14 and 16, September 17) and 2018 (January 18 and February 17) around the road from Urdaneta to Tutupali, which crosses the provinces of Loja, Azuay and Zamora-Chinchipe. The study site (the wetland complex of Oña, Nabón, Saraguro and Yacuambi) has an altitudinal range between 3000 and 3400 m a.s.l. and consists of herb páramo and a wetland complex of almost 100 glacial lakes. We made intensive visual encounter surveys and auditory surveys both during the day and during the night (12h00–02h00). Collected specimens were photographed alive and euthanized using 20% benzocaine, fixed in 10% formalin, and stored in 70% ethanol. Tissue samples for genetic analyses were preserved in 96% ethanol. Examined specimens (listed in the type-series and S1 Appendix) are housed in Museo de Zoología, Universidad Técnica Particular de Loja, Loja, Ecuador (MUTPL), Museo de Zoología, Pontificia Universidad Católica del Ecuador, Quito, Ecuador (QCAZ) and Museo de Zoología, Universidad Tecnológica Indoamérica, Quito, Ecuador (MZUTI).

### Morphology

Taxonomy of *Pristimantis* follows Padial et al. [15] and Brito et al. [14]. The description of qualitative and quantitative morphological characters as well the format of the description follows Duellman and Lehr [16]. Sex was determined by the presence of vocal slits and/or by gonadal inspection. Color data in life were based on field notes and digital photos. The capitalized colors and their corresponding color codes (in parentheses) used in life descriptions follow Köhler [17]. The specimens were weighted (body mass: BM) before euthanasia using a My Weigh Triton T3 portable scale with 0.01 g precision. Measurements were taken with a digital caliper and rounded to the nearest 0.1 mm. All well-preserved specimens were measured for the following morphometric variables: (1) snout-vent length (SVL), distance from tip snout to posterior margin of vent; (2) head width (HW), greatest width of head measured at level of jaw articulation; (3) head length (HL), distance from the tip of snout to posterior angle of jaw articulation; (4) interorbital distance (IOD), distance between the inner margins of the orbits; (5) internarial distance (IND), distance between the inner edges of the narial openings; (6) upper eyelid width (EW), the perpendicular distance to the outer edge of the eyelid; (7) eye diameter (ED), distance between anterior and posterior borders of eye; (8) eye-nostril distance (EN), distance from posterior margin of nostril to anterior margin of eye; (9) tympanum diameter (TD), horizontal distance between peripheral borders of tympanic annulus; (10) femur length (FL), length of femur from vent to knee; (11) tibia length (TL), length of flexed leg from knee to heel; (12) foot length (FoL), distance from proximal margin of inner metatarsal tubercle to tip of Toe IV; (13) hand length (HaL), distance from proximal edge of palmar tubercle to the tip of Finger III.

### DNA extraction, amplification and sequencing

PCR reactions were performed directly from liver tissue using the Extract-N-Amp™ Tissue PCR Kit (Sigma-Aldrich) according to the manufacturer's instructions. The *12S* and *16S* rRNA mitochondrial genes, and the nuclear *RAG-1* gene were amplified using the primers described in S1 Table. Success of PCR amplification was tested by gel electrophoresis, using a 1% agarose gel stained with GelRed™ Nucleic Acid Gel Stain (Biotium). PCR products were purified using 1.1 volumes of PEG (20% Polyethylene glycol 8000, 2.5 M NaCl) followed by incubation at 37°C for 15 min. Samples were then centrifuged at 14000 rpm at room temperature for 15 min, the supernatant was discarded. The DNA pellet was washed twice with ice cold 80% ethanol, spinning the samples at maximum speed in the centrifuge between ethanol washings. The ethanol was discarded and the DNA pellet was dried at room temperature for 5 min or until no ethanol was visible in the microcentrifuge tube. DNA was then resuspended in 15 μL sterile ddH2O. Amplicons were sent for sequencing at Macrogen Sequencing Service (Seoul, Korea) using the corresponding forward primer for each gene. The newly generated DNA sequences were deposited in GenBank (S2 Table).

### DNA sequence analyses

The sequences were edited and assembled using the program Geneious R9 [18], and aligned with the closest BLAST matches downloaded from Genbank using MAFFT online version 7 [19] under the G-INS-i option. For taxon sampling selection we performed a preliminary maximum likelihood phylogenetic analysis including all *12S* and *16S* sequences of *Pristimantis* available from GenBank (489 terminals for *12S* and 1260 terminals for *16S*), using the MEGA 6 software [20]. The resulting trees showed us the position of the new species and, based on this, we used all the available species closest to it for more intensive searches and for calculating support. We included in our analysis sequences from all the available species from the *Pristimantis oreste*s group (as defined by Brito et al. [14]) and 13 closely related species to this group (based on Padial et al. [15]). *Pristimantis galdi* was used to root the tree. The edited alignments of *12S*, *16S* and *RAG-1* sequences were concatenated to get a final single alignment, which was then used for all further phylogenetic analyses. Additionally, we performed separate maximum likelihood phylogenetic analysis for each one of the genes (using the MEGA 6 software) in order to corroborate our concatenated tree. We used PartitionFinder v. 2.1.1 [21] to select the best-fit models of sequence evolution and best partition scheme with the AICc model of selection. Molecular phylogenetic relationships were inferred using Maximum Likelihood (ML) and Bayesian Inference (BI). ML analyses were conducted in GARLI v. 2.1 [22] performing four independent searches (two with the “streefname” set to random and two set to stepwise) with 250 replicates each and with the “genthreshfortopoterm” set to 100,000. Node support was assessed with non-parametric bootstrapping [23] with 1000 pseudoreplicates. The 50% majority rule consensus for the bootstrap trees was obtained with Geneious R9 [18]. BI analyses were conducted with MrBayes 3.2.6 [24], the Markov chain Monte Carlo runs being performed twice, first for 66 million generations and second for 65 million generations, with a sampling frequency of 500. Convergence of the runs was assessed from the average split frequency of standard deviations (*P* < 0.001) and by checking the potential scale reduction factors (PSRF ~ 1.0) for all model parameters. The first 25% of the trees were discarded as burn-in and the remaining ones were used to generate a 50% majority rule consensus tree, as well as to estimate the Bayesian posterior probabilities. Uncorrected *p*-genetic distances for gene 16S were estimated with software MEGA6 [20].

### Call recordings and analysis

We recorded the calls from eleven males in the field using an Olympus LS-11 Linear PCM Recorder and a RØDE NTG2 condenser shotgun microphone at 44.1 kHz sampling frequency and 16-bit resolution, in WAV file format (S3 Table). Air temperature and humidity were measured with a data logger (Lascar Electronics, model EL-USB-2-LCD, accuracy: ± 0.5°C; ± 5%). The original, analyzed call recordings are deposited in full length in the Fonoteca UTPL (records ID are provided in S3 Table). Acoustic analysis was conducted using Raven Pro 1.4 (http://www.birds.cornell.edu/ raven). We measured the temporal parameters from the oscillograms and the spectral parameters from spectrograms obtained through Hanning window function, DFT: 512 samples, 3 dB filter bandwidth: 124 Hz, and 50% overlap.

The terminology and procedures for measuring call parameters follow Cocroft and Ryan [25], Toledo et al. [26] and Köhler et al. [27] and a note-centered approach was used to distinguish between a call and a note (sensu Köhler et al. [27]). The following temporal and spectral parameters were measured and analyzed: (1) *note duration*: the duration of a single note within a call, measured from beginning to the end of the note; (2) *inter-note interval*: the interval between two consecutive notes within the same call, measured from the end of one note to the beginning of the consecutive note; (3) *note rate*: number of notes per second, measured as the time between the beginning of the first note and the beginning of the last note; (4) *dominant frequency*: the frequency containing the highest sound energy, measured along the entire call; and (5) *the 90% bandwidth*, reported as *Frequency 5%* and *Frequency 95%*, or the minimum and maximum frequencies, excluding the 5% below and above the total energy in the selected call. Due to the particularity of the advertisement call (a series of repeated single-pulsed notes) on average 30 notes per call were analyzed.

### Nomenclatural acts

The electronic edition of this article conforms to the requirements of the amended International Code of Zoological Nomenclature (ICZN), and hence the new names contained herein are available under that Code from the electronic edition of this article. This published work and the nomenclatural acts it contains have been registered in ZooBank, the online registration system for the ICZN. The ZooBank LSIDs (Life Science Identifiers) can be resolved and the associated information viewed through any standard web browser by appending the LSID to the prefix “http://zoobank.org/”. The LSID for this publication is: urn:lsid:zoobank.org:pub:2014CE9E-CC5F-4A15-AF68-0F60F321C3ED. The electronic edition of this work was published in a journal with an ISSN, and has been archived and is available from the following digital repositories: PubMed Central and LOCKSS.

## Results

### Phylogeny

**Fig 1:**
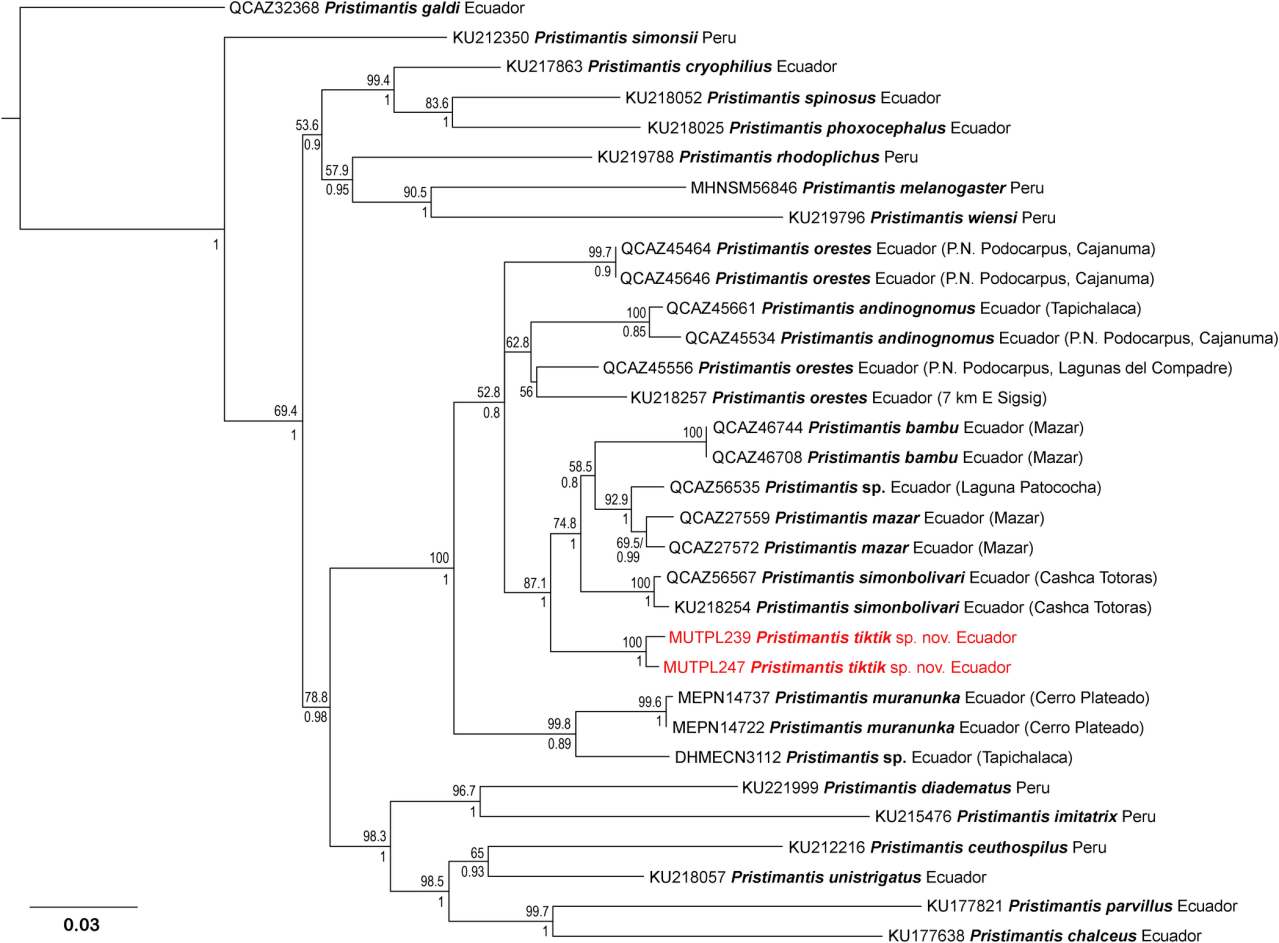
Maximum likelihood phylogram of *Pristimantis tiktik* sp. nov. and related species inferred from 2393 aligned positions (916 positions for *12S*, 896 for *16S* and 581 for *RAG-1* genes). Bootstrap values (%) are shown above the branches and Bayesian posterior probabilities (decimal) are shown below except when they are below 50 (bootstrap) or 0.5 (posterior probability). The tree was rooted with *Pristimantis galdi*. Museum catalog number and country (also locality of origin for vouchers in the case of the species from the *P*. *orestes* group) are shown for each sample (associated data listed in S2 Table).

The final concatenated alignment consisted of 2393 base pairs with 916 positions for *12S*, 896 for *16S* and 581 for *RAG-1*. PartitionFinder under AICc identified three partition schemes as the best strategy (best model in parentheses): *12S* and *16S* (GTR + I + G), *RAG-1* 1^st^ position (HKY + I) and *RAG-1* 2^nd^ and 3^rd^ positions (F81 + I). The phylogenetic trees constructed by Bayesian inference and Maximum likelihood showed the same topology, but with the ML tree providing usually higher support values at deeper nodes (Fig 1). The separate trees for each of the genes showed the same topology with the concatenated one. Both our analyses of *12S* and *16S* and the analysis of the concatenated alignment placed the new species in the *Pristimantis oreste*s group (as defined by Brito et al. [14]), formed by Ecuadorian species. *Pristimantis tiktik* sp. nov. is part of a strongly supported clade in the *P*. *orestes* group and is the sister species of the assemblage that contains *Pristimantis bambu*, *P*. *mazar*, *P*. *simonbolivari* and an undescribed species. Uncorrected p-genetic distances for the gene *16S* between *Pristimantis tiktik* sp. nov. and its closest relatives range from 4.5% to 8.8% (Table 1). Using the conservative 3% threshold of the pairwise genetic uncorrected *p*-distances in the *16S* rRNA gene for species delineation [12] together with the observed morphological and advertisement call differences, we have the reliable confirmation that *Pristimantis tiktik* sp. nov. is an undescribed species, being diagnosed, described and named below.

**Table 1:** Uncorrected pairwise distances (%), for the mitochondrial gene *16S* fragment, among the closest *Pristimantis* species.

		1	2	3	4	5	6	7	8	9	10	11	12	13	14	15
1	*Pristimantis andinognomus* QCAZ45661															
2	*Pristimantis andinognomus* QCAZ45534	1.3														
3	*Pristimantis bambu* QCAZ46744	7.7	7.7													
4	*Pristimantis bambu* QCAZ46708	5.4	5.8	0.0												
5	*Pristimantis mazar* QCAZ27572	6.6	6.8	5.1	4.2											
6	*Pristimantis mazar* QCAZ27559	7.3	7.5	5.4	4.8	1.3										
7	*Pristimantis muranunka* MEPN14722	7.5	7.9	7.0	7.1	7.3	8.0									
8	*Pristimantis muranunka* MEPN14737	8.3	8.5	8.9	6.9	9.0	9.6	0.2								
9	*Pristimantis orestes* KU218257	5.8	6.5	7.6	5.4	6.8	7.4	8.1	8.5							
10	*Pristimantis orestes* QCAZ45556	4.7	5.1	5.1	5.1	4.5	5.5	8.0	7.8	4.0						
11	*Pristimantis simonbolivari* KU218254	6.5	7.3	6.7	4.6	5.1	5.6	6.4	8.4	6.4	3.2					
12	*Pristimantis simonbolivari* QCAZ56567	6.2	6.9	6.2	4.8	4.7	5.3	6.4	8.5	6.5	3.2	0.7				
13	*Pristimantis* sp. QCAZ56535	6.2	6.1	4.2	4.2	2.1	2.7	7.6	7.4	5.5	4.9	3.4	3.8			
14	*Pristimantis* sp. DHMECN3112	8.5	8.7	7.7	6.9	8.4	8.9	4.7	5.6	8.2	7.2	7.8	7.6	7.4		
15	***Pristimantis tiktik* MUTPL239**	**6.9**	**7.0**	**6.8**	**6.1**	**5.4**	**6.2**	**8.3**	**8.8**	**7.0**	**4.9**	**6.0**	**5.7**	**4.9**	**8.5**	
16	***Pristimantis tiktik* MUTPL247**	**6.8**	**6.9**	**6.3**	**5.6**	**5.0**	**5.9**	**8.3**	**8.8**	**6.9**	**4.9**	**6.1**	**5.8**	**4.5**	**8.2**	**0.6**

### Taxonomic treatment

Class Amphibia Linnaeus, 1758

Order Anura Fischer von Waldheim, 1813

Superfamily Brachycephaloidea Günther, 1858

Family Strabomantidae Hedges, Duellman, and Heinicke, 2008

Subfamily Pristimantinae Pyron and Wiens, 2011

Genus *Pristimantis* Jiménez de la Espada, 1870

***Pristimantis tiktik* sp. nov.** Székely, Eguiguren, Székely, Ordóñez-Delgado, Armijos-Ojeda, Riofrío-Guamán, and Cogălniceanu.

urn:lsid:zoobank.org:act:3A4F53A8-74B1-407F-8095-D90CBD98ADCA

(Figs 2–7)

**Fig 2:**
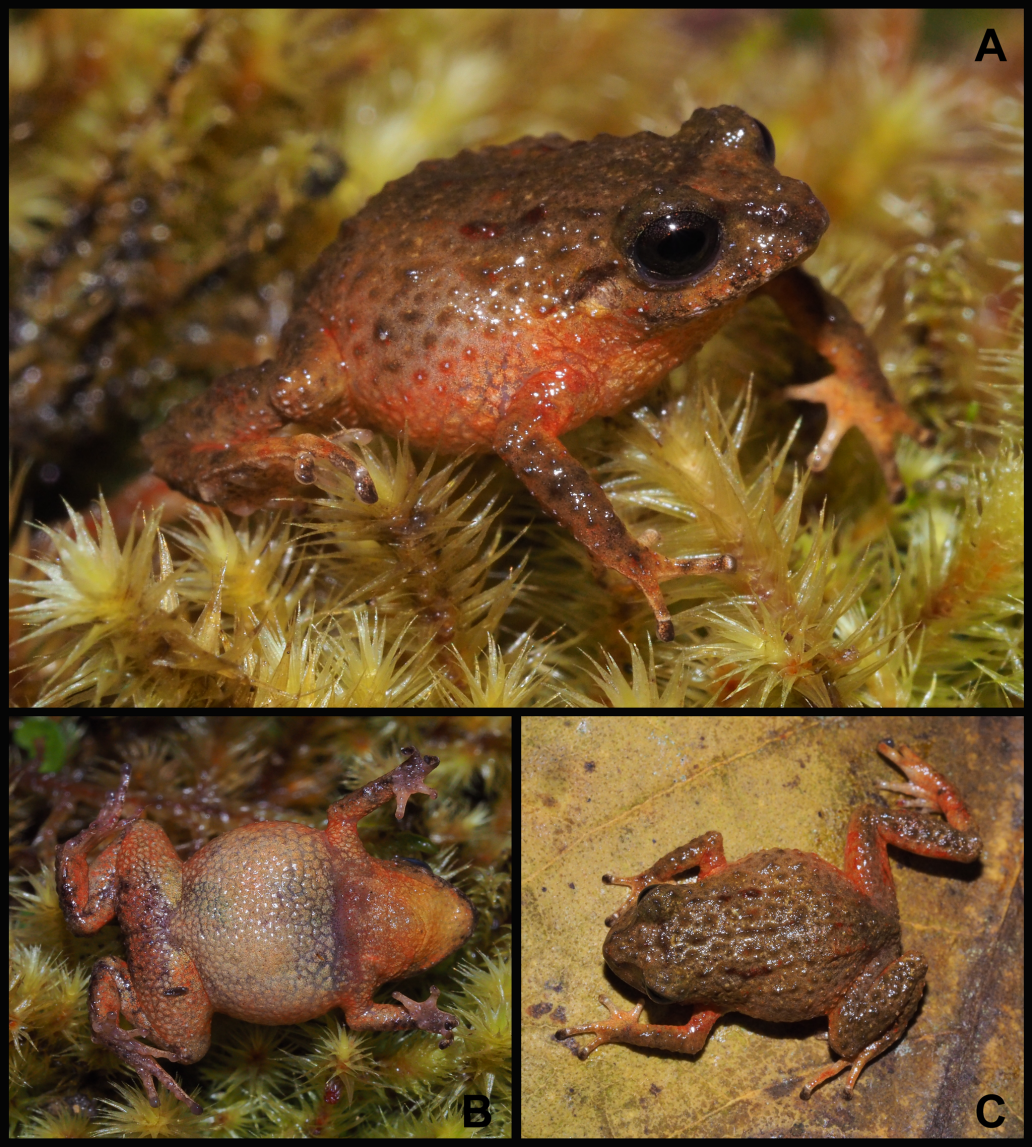
Holotype of *Pristimantis tiktik* sp. nov. (MUTPL 239, adult male), SVL 16.7 mm, in life. **A.** Dorsolateral view; **B.** Ventral view; **C.** Dorsal view.

**Fig 3:**
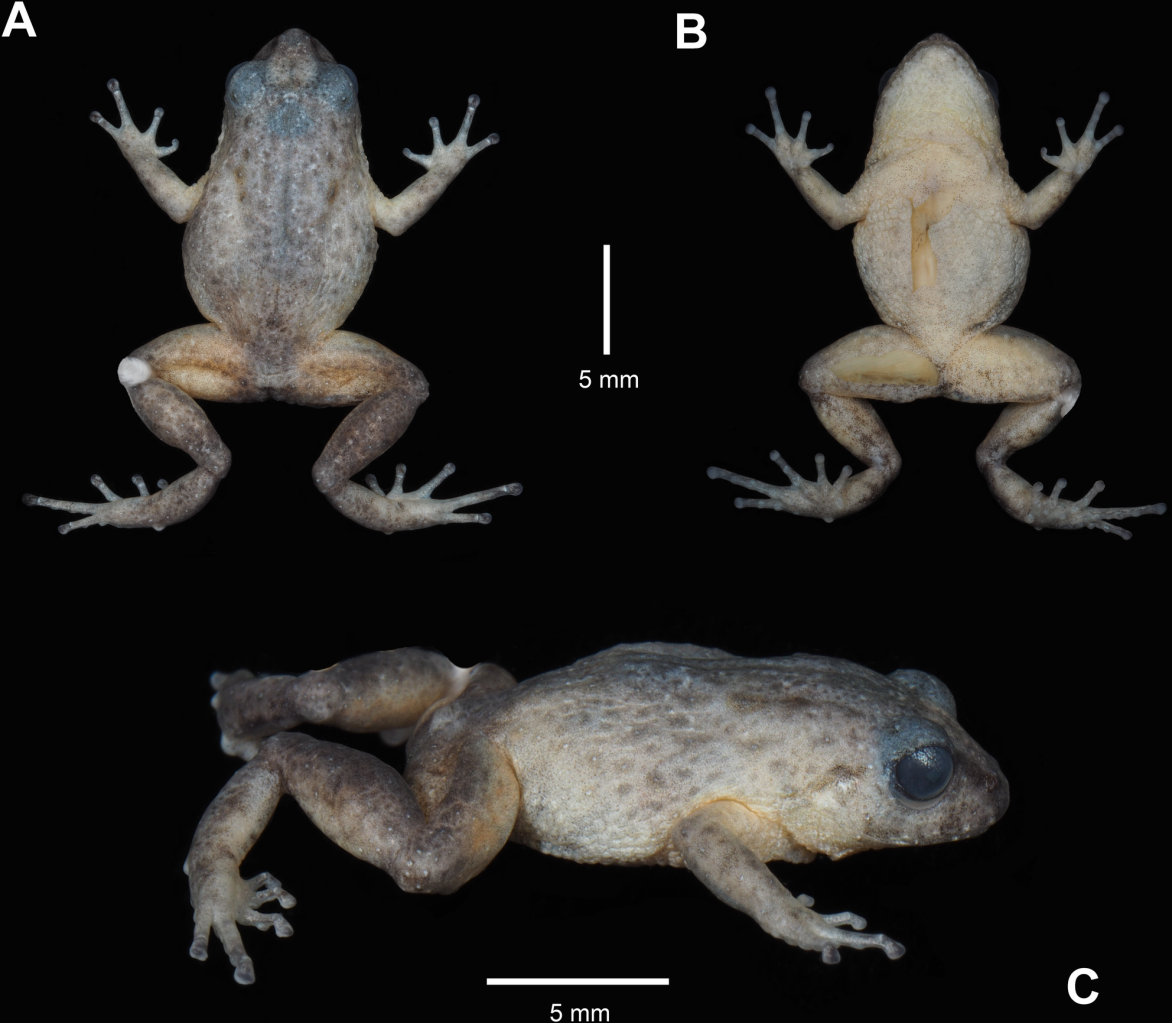
Holotype of *Pristimantis tiktik* sp. nov. (MUTPL 239, adult male) in preservative. **A.** Dorsal view; **B.** Ventral view; **C.** Lateral view.

**Fig 4:**
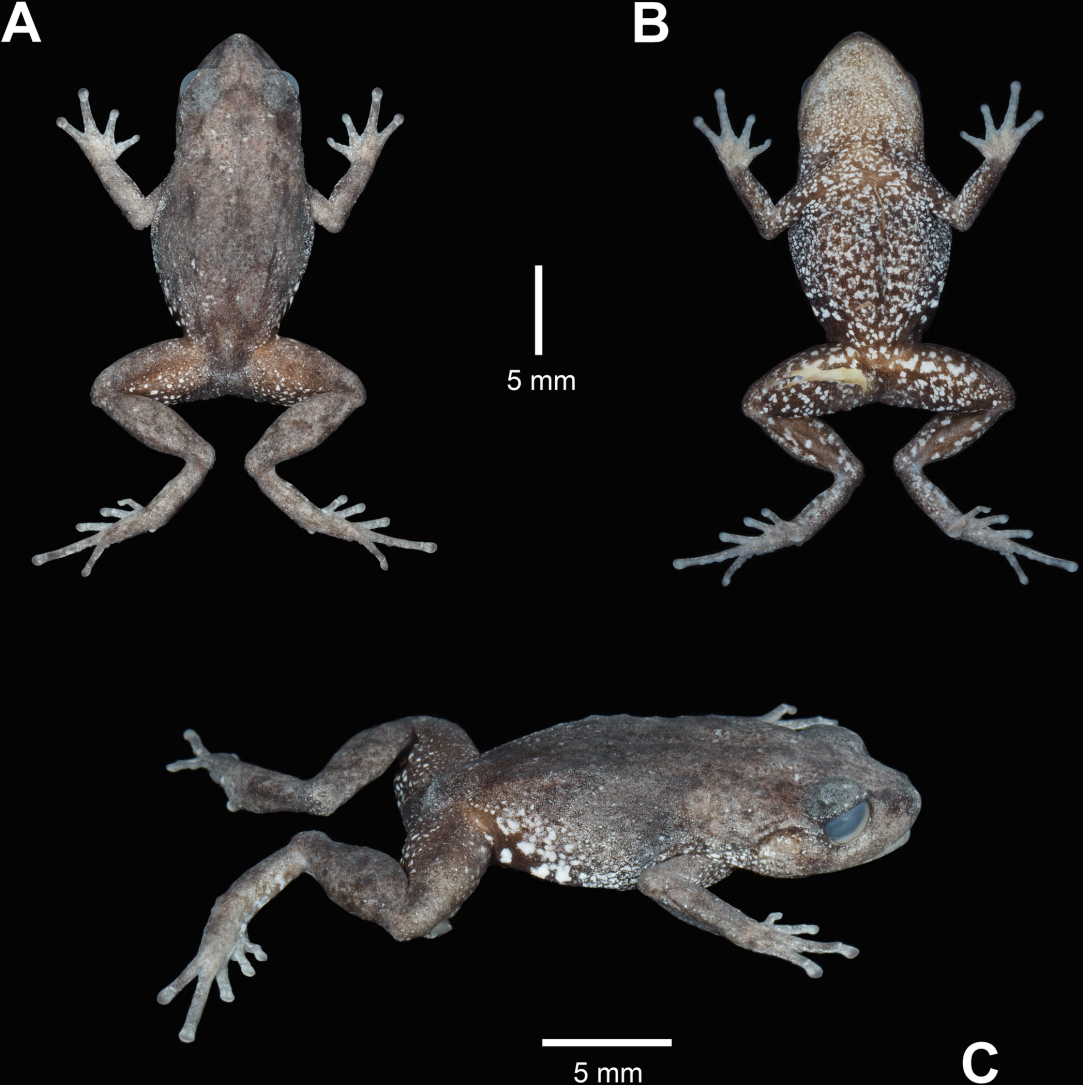
Paratype of *Pristimantis tiktik* sp. nov. (MUTPL 247, adult female) in preservative. **A.** Dorsal view; **B.** Ventral view; **C.** Lateral view.

**Fig 5:**
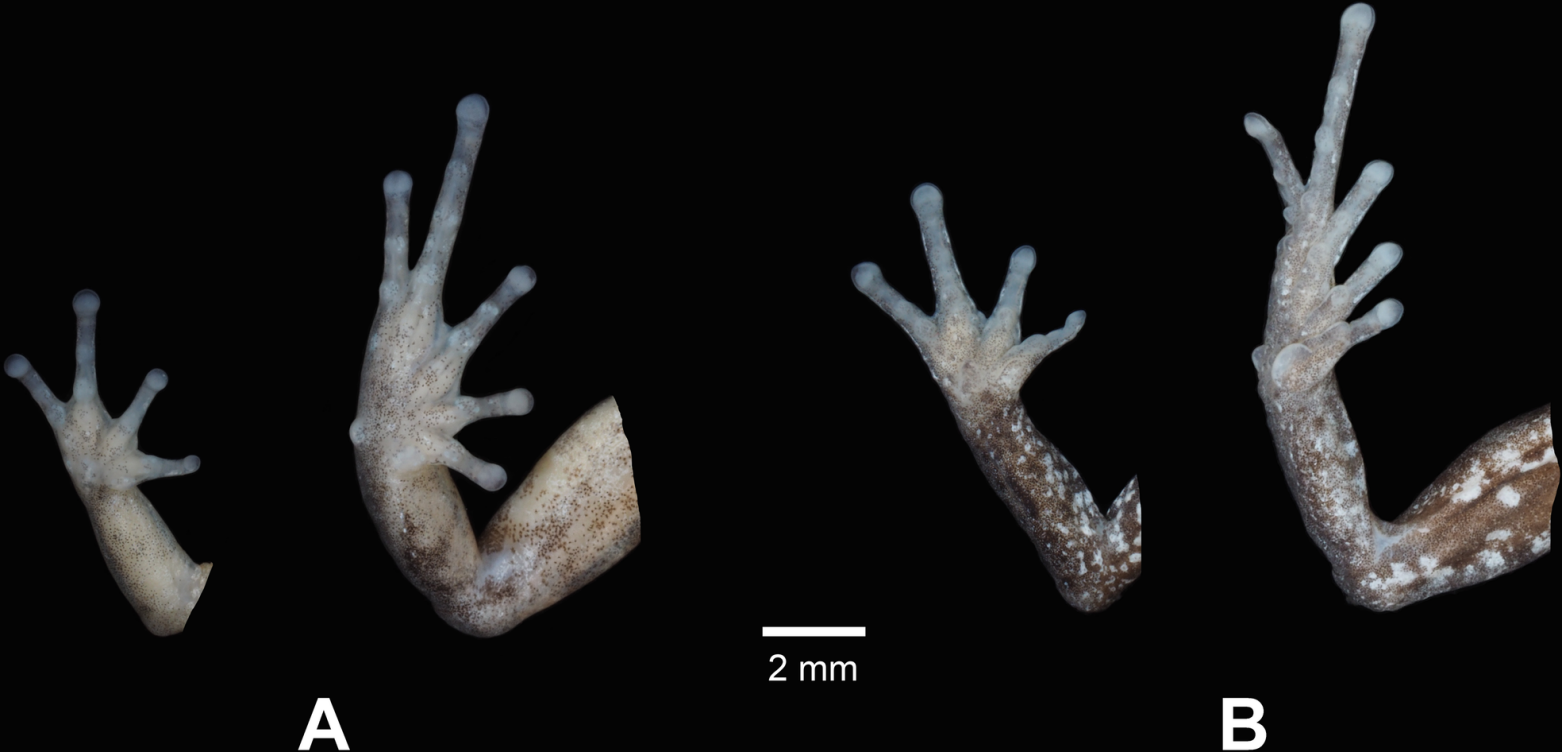
Palmar view of hand and plantar view of foot of A. the holotype of *Pristimantis tiktik* sp. nov. (MUTPL 239, adult male) and B. paratype (MUTPL 247, adult female) in preservative.

**Fig 6:**
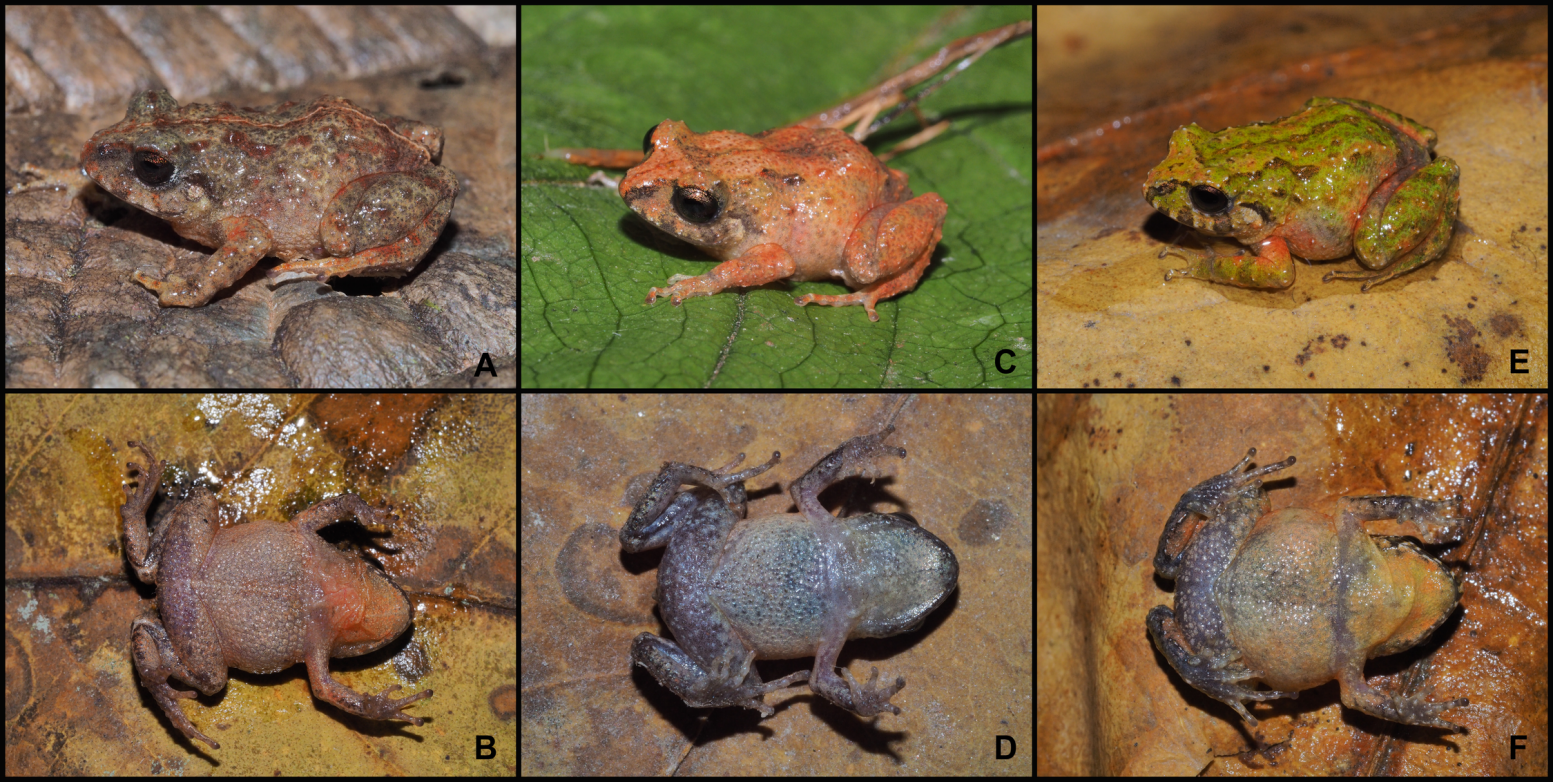
Color variation in males of *Pristimantis tiktik* sp. nov. in life. Paratype (MUTPL 240), SVL 18.4 mm: **A.** dorsolateral view; **B.** ventral view. Paratype (MUTPL 251), SVL 16.1 mm: **C.** dorsolateral view; **D.** ventral view. Paratype (MUTPL 277), SVL 17.0 mm: **E.** dorsolateral view; **F.** ventral view.

**Fig 7:**
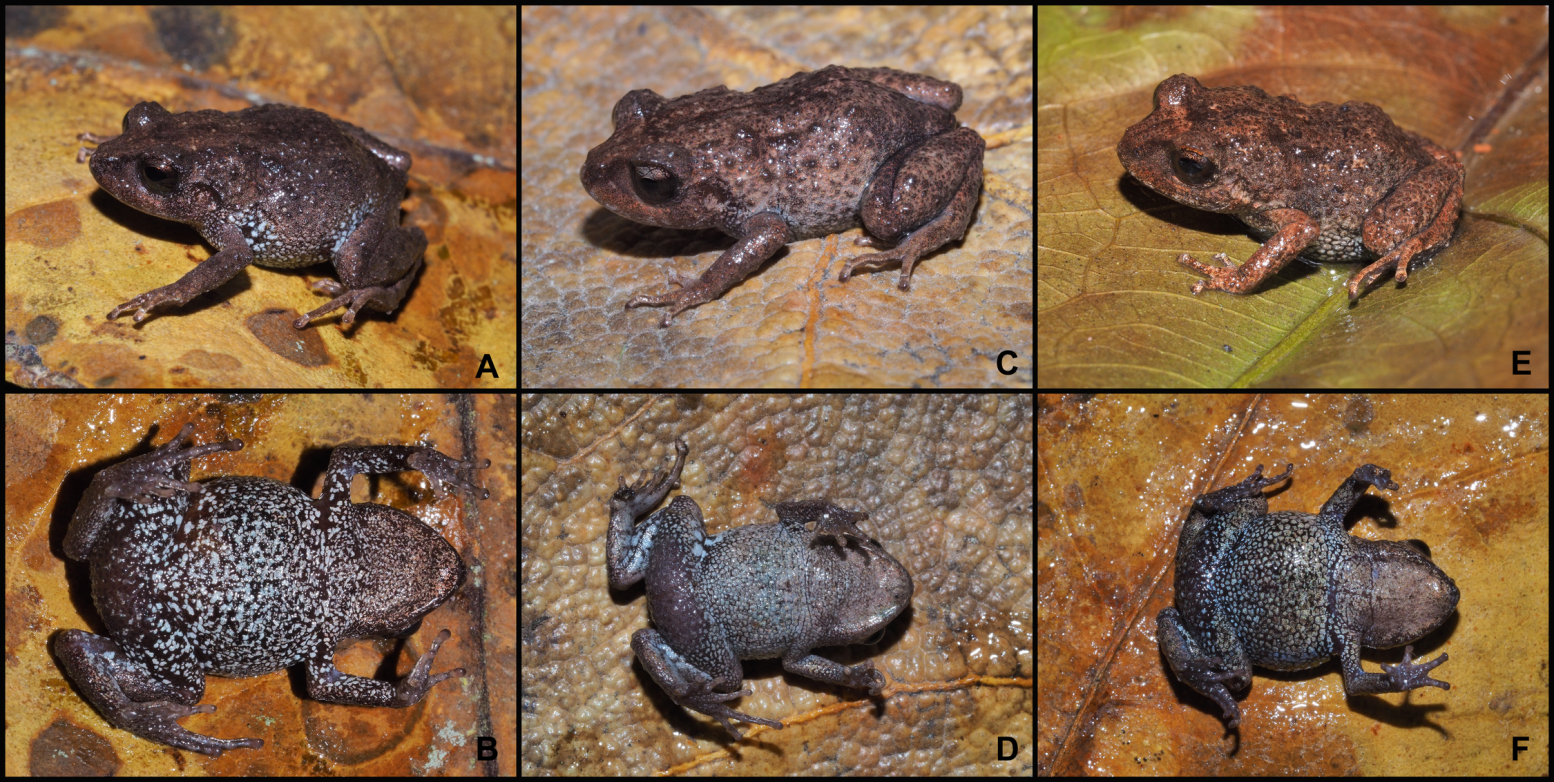
Color variation in females of *Pristimantis tiktik* sp. nov. in life. Paratype (MUTPL 247), SVL 20.2 mm: **A.** dorsolateral view; **B.** ventral view. Paratype (MUTPL 252), SVL 19.7 mm: **C.** dorsolateral view; **D.** ventral view. Paratype (MUTPL 276), SVL 20.4 mm: **E.** dorsolateral view; **F.** ventral view.

**Common English name.** Tiktik Rain Frog

**Common Spanish name.** Cutín tiktik

**Etymology.** The specific name is the onomatopoeic representation of the frog’s particular call.

**Holotype.** MUTPL 239, an adult male (Figs 2, 3 and 5A) from Ecuador, Loja province, Saraguro canton, 21 km (by road) E of Urdaneta (3.58612° S, 79.07516° W; datum WGS84), 3300 m above sea level, collected by Paul Székely, Diego Armijos-Ojeda and Dan Cogălniceanu on 8 July 2016.

**Paratypes (5 males and 3 females).** MUTPL 240, adult male (Fig 6A and 6B), from Ecuador, Loja Province, Saraguro canton, 19 km (by road) E of Urdaneta (3.58055° S, 79.09312° W; datum WGS84), 3296 m above sea level, collected by Paul Székely, Diana Székely, Diego Armijos-Ojeda and Dan Cogălniceanu on 9 July 2016; MUTPL 245 and MUTPL 246, two adult males collected by Paul Székely and Diana Székely on 23 July 2016 at the type locality; MUTPL 247, an adult female (Figs 4, 5B, 7A and 7B) from Ecuador, Loja Province, Saraguro canton, 14 km (by road) E of Urdaneta (3.58554° S, 79.11323° W; datum WGS84), 3108 m above sea level, collected by Paul Székely and Diana Székely on 23 July 2016; MUTPL 251, an adult male (Fig 6C and 6D) and MUTPL 252, an adult female (Fig 7C and 7D) collected by Dan Cogălniceanu and Paul Székely on 4 August 2016; MUTPL 276, an adult female (Fig 7E and 7F) and MUTPL 277, an adult male (Fig 6E and 6F), from Ecuador, Loja Province, Saraguro canton, 17 km (by road) E of Urdaneta (3.56955° S, 79.11258° W; datum WGS84), 3094 m above sea level, collected by Diana Székely and Paul Székely on 14 May 2017.

**Diagnosis.** We assign this species to *Pristimantis* based on phylogenetic evidence (Fig 1) and on the general morphological similarity to other members of the genus. *Pristimantis tiktik* is a small species distinguished by the following combination of traits: (1) skin on dorsum tuberculated; skin on venter coarsely areolate; discoidal fold weak, more evident posteriorly; thoracic fold absent; dorsolateral folds absent; low mid dorsal fold present; (2) tympanic membrane absent but tympanic annulus evident, its length about 30% of the length of eye; supratympanic fold present; (3) snout short, subacuminate in dorsal view, rounded in profile; canthus rostralis weakly concave in dorsal view, rounded in profile; (4) upper eyelid bearing several small tubercles, similar in size and shape with the ones from the dorsum, about 80% IOD in females and 70% IOD in males; cranial crests absent; (5) dentigerous processes of vomers inconspicuous, elongated, but each processes bearing 3 to 5 evident teeth; (6) males with a large subgular vocal sac, extended onto the chest; vocal slits present; nuptial pads absent; (7) Finger I shorter than Finger II; discs on fingers just slightly expanded, rounded; circumferential grooves present; (8) fingers bearing narrow lateral fringes; subarticular tubercles prominent; supernumerary palmar tubercles present, rounded, smaller than subarticular tubercles; palmar tubercle inconspicuous, bifurcated; thenar tubercle oval; (9) ulnar tubercles coalesced into low ulnar fold; (10) heel lacking enlarged tubercles; outer edge of tarsus with row of small tubercles; inner edge of tarsus bearing a long fold; (11) inner metatarsal tubercle broadly ovoid, about 3x round outer metatarsal tubercle; supernumerary plantar tubercles present; (12) toes bearing narrow lateral fringes; webbing absent; Toe V slightly longer than Toe III; discs on toes just slightly expanded, rounded, about same size as those on fingers; circumferential grooves present; (13) evident sexual dimorphism: in life, the males with dorsum of various shades of gray, brown, orange or green (brownish gray or gray in females), the flanks, chest, groins and ventral surface of the limbs have usually a reddish mottling and the venter is whitish or pinkish yellow (venter, axillae and groins white with black reticulum in females); iris bronze, with lower half darker, and with fine black reticulations; (14) SVL 19.7–20.4 mm in adult females (20.1 ± 0.36 SD, n = 3) and 16.1–18.4 mm in adult males (16.9 ± 0.79 SD, n = 6).

**Comparisons with similar species.** *Pristimantis tiktik* is morphologically similar to its closest relatives, the species from the recently redefined *P*. *orestes* group [14], but its characteristic morphological features easily distinguish it from all the resembling species. The phylogenetically closest species are *P*. *simonbolivari*, *P*. *bambu* and *P*. *mazar* (Fig 1). However, *P*. *simonbolivari* (females up to 27 mm, males up to 21 mm; QCAZ 16823, 16925), *P*. *bambu* (females up to 27 mm, males up to 20 mm; QCAZ 68141, 68402) and *P*. *mazar* (females up to 23 mm, males up to 18 mm; [28]) are significantly larger and neither one has tuberculated skin on dorsum as *P*. *tiktik*. Additionally, *P*. *tiktik* does not have the characteristic large white spots on a black background of the groin, thighs and shanks present in *P*. *simonbolivari*, the similar large yellow spots present in *P*. *bambu* or the characteristic black spots on the groin and ventral coloration with the reticulated pattern of *P*. *mazar*.

*Pristimantis orestes* (females up to 27 mm, males up to 23 mm; QCAZ 58569, [29]) is also significantly larger, does not have the tuberculated skin on dorsum as *P*. *tiktik* and has characteristic large white spots on a black background of the groin, thighs and shanks (absent in *P*. *tiktik*). *Pristimantis andinognomus* is smaller (females up to 17 mm, males up to 14 mm; [30]) than *P*. *tiktik*, has the skin on dorsum shagreen (tuberculated in *P*. *tiktik*), Toe V much longer than Toe III (Toe V slightly longer than Toe III in *P*. *tiktik*) and the groin, anterior surfaces of thighs, and concealed surfaces of shanks are tan with minute brown spots (absent in *P*. *tiktik*). *Pristimantis muranunka* is easily distinguished from *P*. *tiktik* by the shagreen dorsum (tuberculated in *P*. *tiktik*), the much smaller fingers than those of *P*. *tiktik* and the general blackish dorsal and ventral coloration.

Among the Ecuadorian species that possibly belong to *P*. *orestes* group (but for now their phylogenetic position is still unknown), only two resemble, to some extent, *P*. *tiktik*: *P*. *saturninoi* and *P*. *tinajillas*. However, *P*. *saturninoi* has dorsolateral folds (absent in *P*. *tiktik*), has a similar blackish coloration of the dorsum and the venter (venter different in *P*. *tiktik*), the groin, thighs and shanks black with whitish or yellowish spots (absent in *P*. *tiktik*) and has green iris (bronze in *P*. *tiktik*). *Pristimantis tinajillas* has shagreen dorsum (tuberculated in *P*. *tiktik*), males with red spots in the groin (absent in *P*. *tiktik*) and unique cuspidate finger and toe discs (rounded in *P*. *tiktik*).

As for the northern Peruvian species with unresolved phylogenetic position that could be members of the *P*. *orestes* group (as defined by Brito et al. [14]) and are somewhat similar to *P*. *tiktik* (*P*. *atrabracus*, *P*. *chimu*, *P*. *pinguis*, and *P*. *stictoboubonus*) neither one of them has the tuberculated skin on dorsum of the *P*. *tiktik* and all are significantly larger. Another similar Peruvian species but with tuberculated dorsum, *P*. *cordovae*, differs from *P*. *tiktik* by its significantly larger size (females up to 23 mm, males up to 27 mm; [16,31]), emarginated (notched) discs on fingers and toes and by the orange blotches or stripes in the groin and on the anterior and posterior surfaces of the thighs (absent in *P*. *tiktik*).

And finally, neither one of the aforementioned Ecuadorian species has an advertisement call like that of *P*. *tiktik*, which consists of clicking “tik” notes emitted in a continuous sequence.

**Description of the holotype.** Adult male (MUTPL 239; Figs 2, 3 and 5A), head narrower than body, wider than long, head length 96% of head width, head width 37% of SVL; head length 36% of SVL; snout short (snout to eye distance 16% of SVL), subacuminate in dorsal view, rounded in profile; canthus rostralis weakly concave in dorsal view, rounded in profile; loreal region flat; eye diameter notably greater than eye-nostril distance; nostrils slightly protuberant laterally; lips not flared; cranial crests absent; upper eyelid bearing several small tubercles, similar in size and shape with the ones from the dorsum, width of upper eyelid 76% of IOD; half of tympanic annulus evident, round, its upper and posterodorsal part obscured by rounded supratympanic fold; tympanic membrane absent; diameter of tympanum 27% of the length of eye; one larger postrictal tubercle situated posteroventrally to tympanic annulus; choanae not visible; dentigerous processes of vomers inconspicuous, elongated, each processes bearing 3 to 4 teeth; tongue 3X as long as wide, slightly notched posteriorly, posterior half not adherent to floor of mouth; large subgular vocal sac (Fig 2B), extended onto the chest; vocal slits slightly concave, located at posterior half of mouth floor in between tongue and margin of jaw; nuptial pads absent.

Skin on dorsum tuberculated with numerous small tubercles; thin, low mid dorsal fold starting at tip of snout and ending at cloaca (trait more visible in life, Fig 2C); dorsolateral folds absent; skin on throat, chest, belly, and ventral surfaces of thighs coarsely areolate; discoidal fold weak, more evident posteriorly; ornamentation in cloacal region absent.

Ulnar tubercles present, coalescing into low ulnar fold (trait more visible in life, Fig 2A and 2B); outer palmar tubercle inconspicuous, bifurcated; thenar tubercle oval; subarticular tubercles prominent, round and subconical in section; supernumerary palmar tubercles rounded, smaller than subarticular tubercles; fingers bearing narrow lateral fringes; relative length of fingers I < II < IV < III; discs on fingers just slightly expanded, rounded; all fingers bearing pads well defined by circumferential grooves (Fig 5A).

Hind limbs short; tibia length 42% of SVL; foot length 41% of SVL; heel lacking enlarged tubercles; outer edge of tarsus with row of small tubercles (trait more visible in life); inner edge of tarsus bearing a long fold; inner metatarsal tubercle broadly ovoid, about 3x round and conical outer metatarsal tubercle; subarticular tubercles prominent, round and subconical in section; plantar supernumerary tubercles rounded, smaller than subarticular tubercles; toes bearing narrow lateral fringes; webbing absent; discs on toes just slightly expanded, rounded, about same size as those on fingers; toes with ventral pads well defined by circumferential grooves; relative length of toes I <II < III < V < IV (Fig 5A); Toe V slightly longer than Toe III (tip of Toe III not reaching the penultimate subarticular tubercle on Toe IV, tip of Toe V not reaching the proximal edge of distal subarticular tubercle on Toe IV).

**Coloration of holotype.** In life (Fig 2): dorsum brownish gray (Dark Drab—45) with some of the tubercles dark red (Dark Carmine—61); flanks, chest, groin and ventral surface of the limbs lighter, pale yellow (Pale Sulphur Yellow—92) with reddish orange (Chrome Orange—74) mottling; venter whitish yellow (Chamois—84) and the throat yellow (Trogon Yellow—81); iris bronze, with lower half darker, and with fine black reticulations.

In preservative (Fig 3): dorsal coloration brownish gray; the coloration of flanks, groin and ventral surface of the limbs became whitish cream with the reddish orange mottling transformed in pale orange; venter and throat whitish cream.

**Measurements of holotype (in mm):** SVL 16.7; head width 6.2; head length 6.0; IOD 2.1; internarial distance 1.6; upper eyelid width 1.6; eye diameter 2.2; eye-nostril distance 1.3; snout to eye distance 2.6; eye to tympanum distance 0.7; tympanum diameter 0.6; femur length 7.1; tibia length 7.0; foot length 6.9; hand length 4.0; Finger I length 1.9.

**Body mass of holotype:** 0.43 g.

**Variation.** Morphometric variability is described in Table 2 and S4 Table. This species displays an evident sexual dimorphism. The males (Fig 6) are smaller and have more vivid colors than the females (Fig 7). The dorsal coloration of the males varies from gray (Drab-Gray—256; MUTPL 240, Fig 6A), brownish gray (Dark Drab—45; MUTPL 239, Fig 2), reddish orange (Peach Red—70; MUTPL 251, Fig 6C) to yellow (Cream Yellow—82; MUTPL 246) and various shades of green (Light Yellow-Green—100, Parrot Green—121 or Chartreuse—89; MUTPL 245, 277, Fig 6E). In many males the flanks, chest, groins and ventral surface of the limbs have a reddish orange (Chrome Orange—74) mottling (MUTPL 240, 245, 246, 277, Figs 2 and 6E). The venter is whitish yellow (Chamois—84; MUTPL 239, 245, 251, 277, Figs 2, 6D and 6F) or pinkish yellow (Light Flesh Color—250; MUTPL 240, 246, Fig 6B). The females are larger and the dorsal coloration is only brownish gray (Raw Umber—22; MUTPL 276, Fig 7E) or gray (Light Neutral Gray—297 or Medium Neutral Gray—298; MUTPL 247, 252, Fig 7A and 7C). The venter is white with black reticulum (Fig 7B, 7D and 7F), a feature that was not observed in neither one of the encountered males (more than 20 individuals). The axillae and groins are also white with black reticulum but the black parts are larger than the ones from the venter (Fig 7A and 7B). In some of the males the low mid dorsal fold has a different, usually lighter, coloration than the rest of the dorsum, making it visible from a distance (Fig 6A).

**Table 2:** Body mass (in grams), measurements (in mm) and morphological proportions (in percentages) of adult females and males of *Pristimantis tiktik* sp. nov. Values are given as range (average ± SD). Female body mass includes eggs.

Character	females (n = 3)	males (n = 6)
Body mass (BM)	0.88–0.94 (0.91 ± 0.03)	0.43–0.70 (0.56 ± 0.11)
Snout-vent length (SVL)	19.7–20.4 (20.1 ± 0.36)	16.1–18.4 (16.9 ± 0.79)
Head width (HW)	6.9–7.2 (7.1 ± 0.15)	6.0–7.1 (6.3 ± 0.39)
Head length (HL)	6.1–6.4 (6.2 ± 0.15)	5.7–6.3 (6.0 ± 0.19)
Interorbital distance (IOD)	2.1–2.1 (2.1 ± 0)	2.0–2.2 (2.1 ± 0.08)
Internarial distance (IND)	1.6–1.6 (1.6 ± 0)	1.3–1.6 (1.5 ± 0.11)
Upper eyelid width (EW)	1.6–1.8 (1.7 ± 0.12)	1.4–1.7 (1.5 ± 0.13)
Eye diameter (ED)	2.2–2.3 (2.2 ± 0.06)	1.9–2.2 (2.1 ± 0.15)
Eye-nostril distance (EN)	1.5–1.7 (1.6 ± 0.12)	1.2–1.5 (1.3 ± 0.10)
Tympanum diameter (TD)	0.7–0.9 (0.8 ± 0.10)	0.5–0.8 (0.6 ± 0.10)
Femur length (FL)	7.9–8.2 (8.0 ± 0.17)	6.9–7.8 (7.3 ± 0.32)
Tibia length (TL)	8.0–8.1 (8.1 ± 0.06)	7.0–7.8 (7.4 ± 0.33)
Foot length (FoL)	7.0–7.7 (7.4 ± 0.36)	6.2–7.5 (6.8 ± 0.51)
Hand length (HaL)	4.4–4.5 (4.4 ± 0.06)	3.6–4.6 (4.0 ± 0.35)
HW/SVL	34.2–36.5	36.4–38.6
HL/SVL	30.4–31.7	34.2–35.9
HL/HW	84.7–92.8	88.7–98.3
EN/HL	24.2–27.9	20.3–23.8
ED/HL	34.4–37.1	31.7–36.8
EW/IOD	76.2–85.7	66.7–77.3
EN/ED	65.2–77.3	59.1–73.7
TD/ED	31.8–40.9	26.3–36.4
FL/SVL	39.1–40.2	41.8–44.7
TL/SVL	39.7–40.6	41.9–45.3
FoL/SVL	35.5–38.1	37.6–42.0

**Fig 8:**
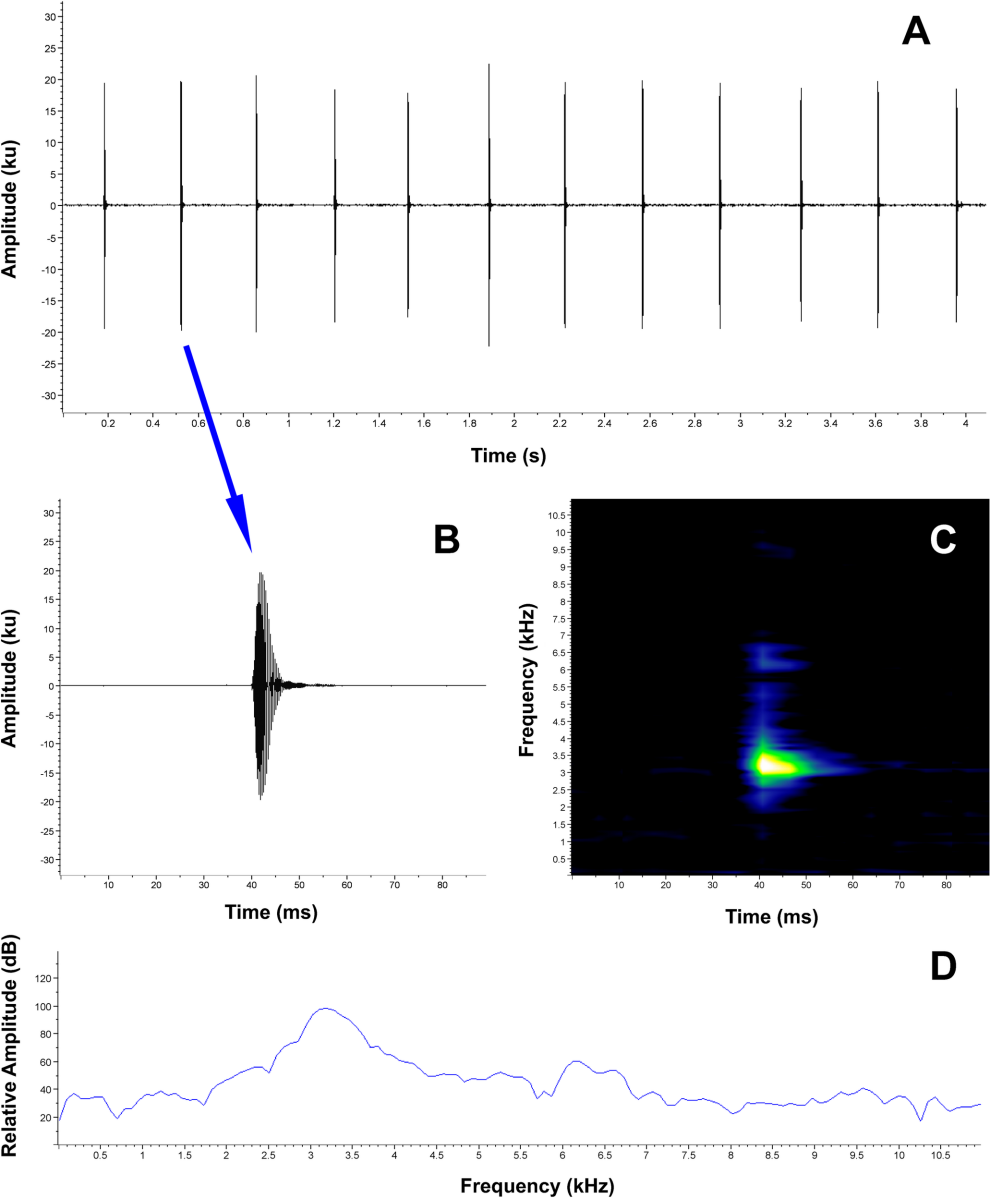
Advertisement call of *Pristimantis tiktik* sp. nov. (holotype, MUTPL 239). **A.** Oscillogram of a 12 notes section of the call; **B.** Oscillogram of a single note; **C.** Spectrogram of a single note; **D.** Power spectrum of a single note.

**Advertisement call.** The advertisement calls of eleven males was recorded between 2016 and 2018 (the detailed information of each of the separate recordings is presented in the S3 Table). *Pristimantis tiktik* has a very particular advertisement call (Fig 8; S1 File) composed by clicking, “tik”, notes which are repeated continuously, like a clock. This distinct call was the inspiration for the species name. It is a simple call composed only of short, single-pulsed notes. Because the males are calling continuously for large intervals, the call duration is unknown. Our longest recording (LS110344, MUTPL 246) lasts for a continuous 6 minutes but very probable the males are able to call for a much longer period of time. The calls are characterized by notes (range values and mean ± SD are provided for all acoustic parameters analyzed) with a duration of 0.007–0.024 s (0.013 ± 0.004, n = 330), an inter-note interval of 0.225–0.354 s (0.287 ± 0.029, n = 319) and a note rate of 2.8–4.0 notes/s (3.36 ± 0.318, n = 11). The 90% bandwidth ranged from 2842.4–3273.0 Hz (3059.5 ± 112.207, n = 330) to 3186.9–3617.6 Hz (3390.5 ± 86.571, n = 330), with the dominant frequency being at 3014.6–3359.2 Hz (3190.8 ± 99.873, n = 330). The fundamental frequency is not recognizable but 3 to 4 harmonics are usually visible. The bioacoustic measurements for each of the recorded males are presented separately in the S3 Table.

**Fig 9:**
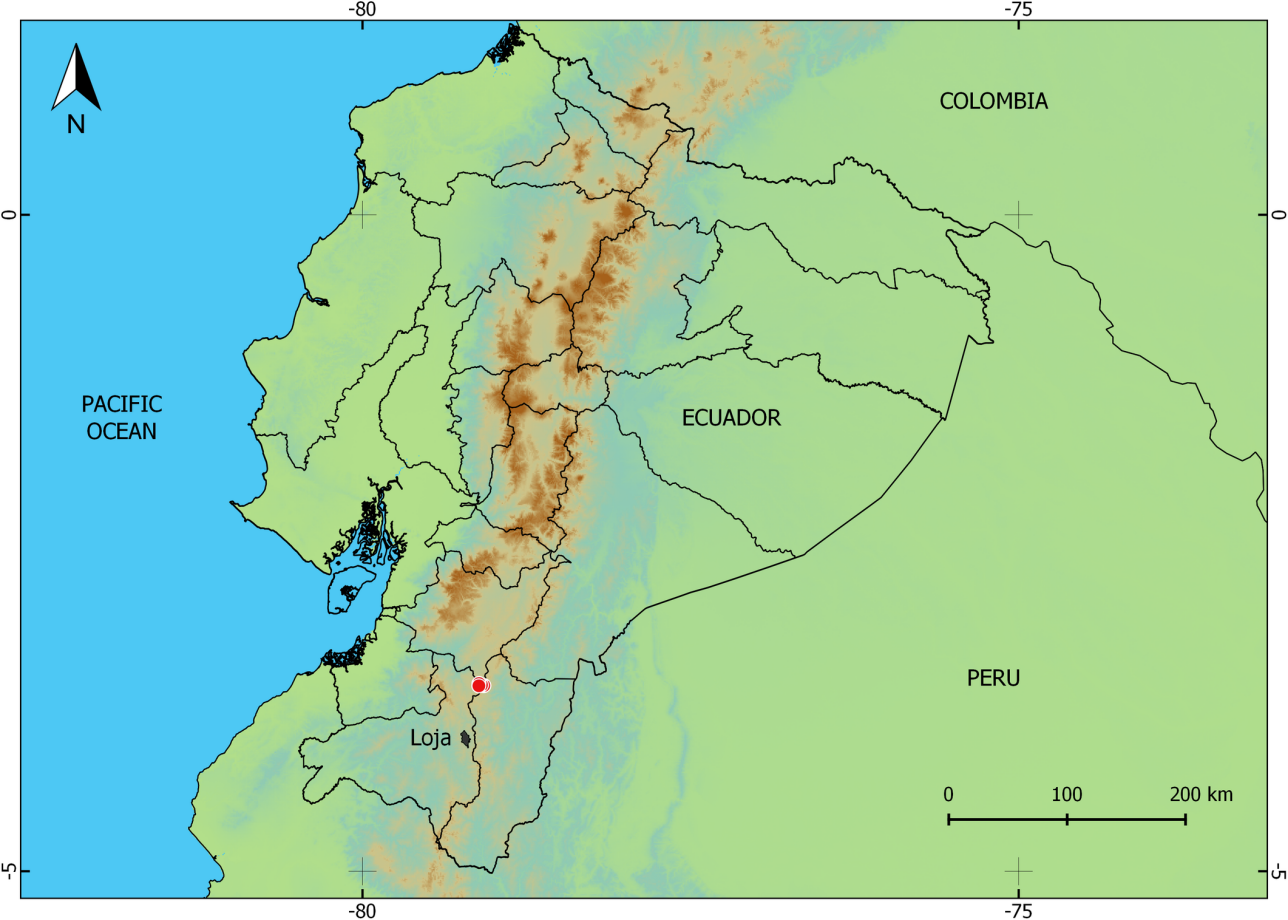
Distribution of *Pristimantis tiktik* sp. nov. (red dots) in Ecuador.

**Fig 10:**
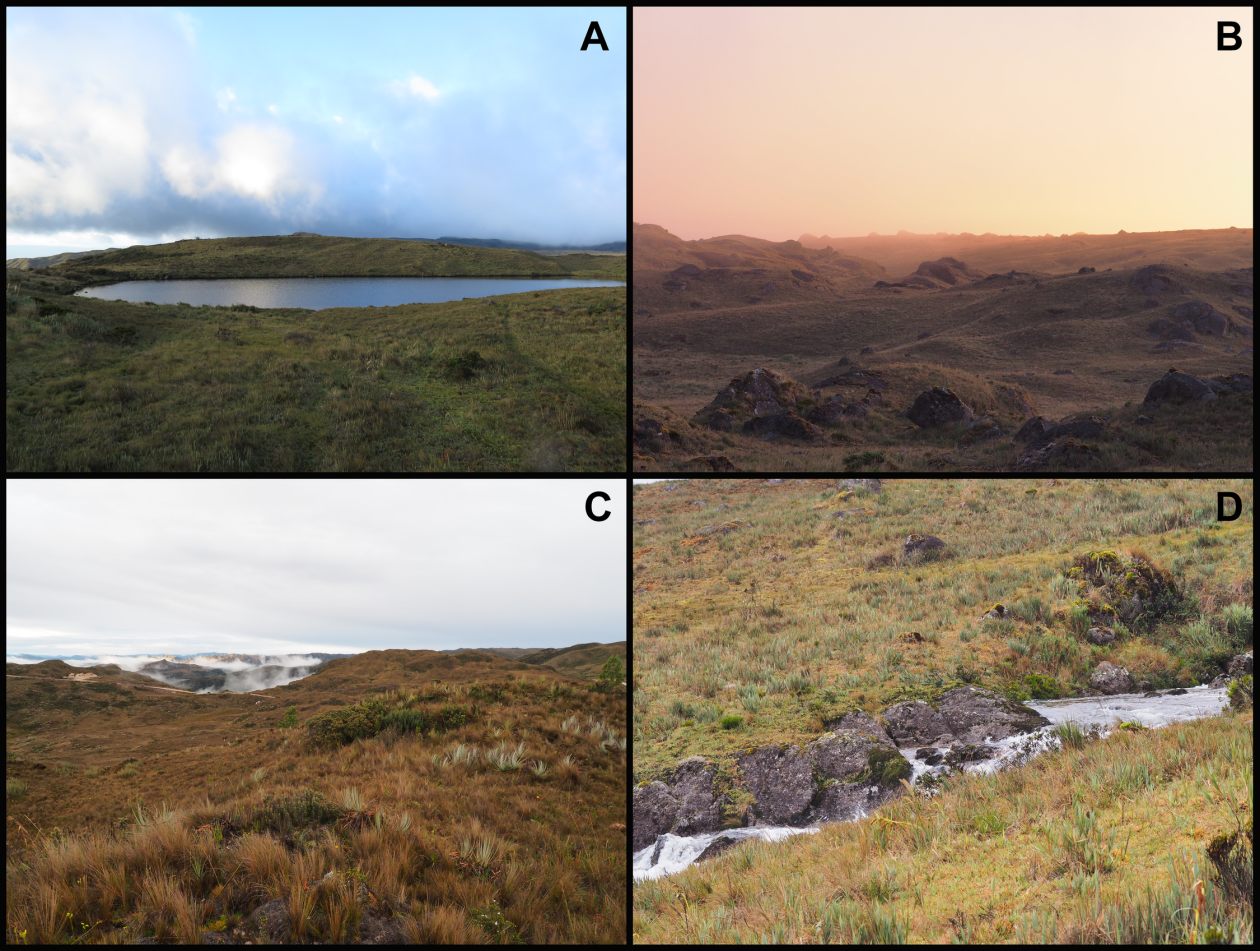
Habitat of *Pristimantis tiktik* sp. nov. in the wetland complex of Oña, Nabón, Saraguro and Yacuambi. **A.** One of the many glacial lakes from the wetland complex; **B.** General view of the herb páramo (montane grassland); **C.** Microhabitat with grasses and shrubs; **D.** Grass microhabitat near a stream from the wetland complex.

**Distribution.** *Pristimantis tiktik* is known only from the wetland complex of Oña, Nabón, Saraguro and Yacuambi (Fig 9) which spreads over three provinces, Loja, Azuay and Zamora-Chinchipe, in Southern Ecuador. This area has an altitudinal range between 3000 and 3400 m a.s.l. and consists of herb páramo (montane grasslands and shrublands) and a wetland complex of almost 100 glacial lakes (Fig 10). We found this species above 3000 m along the road that crosses this area from Urdaneta to Tutupali, but it is probably widespread in the entire wetland complex.

**Natural history.** All the specimens were encountered during the night on the grassy vegetation, very close to the ground (usually at 5–15 cm above the ground). The distinctive call of the males was heard throughout the year (usually after 18:00), regardless of the weather conditions, i.e. rain or strong winds. All the females were caught in the vicinity of the calling males. This seems to be one of the most common frog species from the wetland complex, along with *Pristimantis* aff. *riveti*. Other sympatric frog species include *Gastrotheca pseustes* and a currently undescribed species of *Pristimantis*.

**Conservation status.** *Pristimantis tiktik* is known only from the wetland complex of Oña, Nabón, Saraguro and Yacuambi, above 3000 m a.s.l., which is estimated to have an area of 192 km^2^. Even though this is one of the most commonly encountered species in the wetland complex, we consider it to be Endangered following B1ab(i,ii,iii)+2ab(i,ii,iii) IUCN criteria [32] because: (1) its Extent of occurrence (EOO) and Area of occupancy (AOO) are estimated to be less than 200 km^2^; (2) it is known from only one location; and (3) its habitat is currently affected (or could be severely affected in the near future) by mining activities, invasive species (especially pines from the nearby pine plantations), grazing, wildfires and road constructions.

## Discussion

### Systematics

The small species of the *Pristimantis orestes* group were first recognized as a distinct assemblage by Lynch and Duellman [33], who included three Ecuadorian species (*P*. *orestes*, *P*. *simonbolivari* and *P*. *vidua*). In 2008, Hedges et al. [34] redefined the group and added 11 Peruvian species to the existing ones from Ecuador: *P*. *atrabracus*, *P*. *chimu*, *P*. *cordovae*, *P*. *corrugatus*, *P*. *melanogaster*, *P*. *pataikos*, *P*. *pinguis*, *P*. *seorsus*, *P*. *simonsii*, *P*. *stictoboubonus*, and *P*. *ventriguttatus*. The monophyly of this group was rejected by Pinto-Sánchez et al. [35] and Padial et al. [15], who showed that *P*. *melanogaster* and *P*. *simonsii* are not part of the group, using in their phylogenetic analyses sequences from the only four species available at that time: *P*. *melanogaster*, *P*. *orestes*, *P*. *simonbolivari*, and *P*. *simonsii*. However, in 2017, Brito et al. [14] resurrected the group for the Ecuadorian species, including six described species (*P*. *andinognomus*, *P*. *bambu*, *P*. *mazar*, *P*. *muranunka*, *P*. *orestes*, and *P*. *simonbolivari*) and two undescribed ones, all with the morphological features consistent with the definition of the *P*. *orestes* group as presented by Hedges et al. [34]: small size, short snouts, robust bodies, relative narrow heads, proportionately short limbs and narrow and rounded digital discs.

Our phylogenetic analysis recovered the main subdivisions obtained by Padial et al. [15] and Brito et al. [14] with a strong support, even with higher BI values compared with the results of Brito et al. [14] for the newly defined *P*. *orestes* group (Fig 1). Our analysis also shows that *P*. *melanogaster* and *P*. *simonsii* are not part of the resurrected *P*. *orestes* group. The main differences with the phylogenetic tree of Brito et al. [14] refers to the position of species currently assigned as *P*. *orestes* (possibly three different species) and *P*. *andinognomus*, whose relations to the other species of the group was not resolved in either one of the studies (low support for both ML and BI). The relationships of these species with the other members of the group will probably change with additional sequence data and species sampling.

*Pristimantis tiktik* is part of a strongly supported clade and is the sister species of the assemblage that contains *P*. *bambu*, *P*. *mazar*, *P*. *simonbolivari* and an undescribed species (Fig 1). The exact positions of the species within this assemblage was not resolved (similar to the results of Brito et al. [14]) but the whole subgroup got strong support for both ML and BI. It is worth mentioning that *P*. *tiktik* it seems to be part of a clade of significantly smaller sized species that have the same type of continuous advertisement calls and tuberculated dorsum based on our data (we recently discovered two similar species whose description is currently in preparation).

Finally, it is clear that *P*. *melanogaster* and *P*. *simonsii* are not part of the redefined *P*. *orestes* group and it is imperative to get the molecular data from all the other species from the group of Hedges et al. [34] in order to clarify the phylogenetic relationships of these fascinating species and confirm or not the inclusion of the missing Peruvian species in the new *P*. *orestes* group.

### Conservation remarks

The wetland complex of Oña, Nabón, Saraguro and Yacuambi is in the process of becoming a Ramsar site due to its ecological importance; it houses several endangered and vulnerable species with special ecological requirements such as Andean Tapir (*Tapirus pinchaque*), Andean Bear (*Tremarctos ornatus*) and the Andean condor (*Vultur gryphus*). Unfortunately, even if some of its area is part of the Yacuambi Community Reserve, the wetland complex currently doesn’t benefit from efficient, national level, protection measures, and this allows the development of activities that severely affect this ecosystem (construction of infrastructure without any environmental consideration, manmade wildfires followed by intensive cattle grazing, mining activities). Additionally, the wetland complex currently is affected by the introduction of invasive species such as pine trees and Rainbow Trout (*Oncorhynchus mykiss*).

The extension of the nearby mining activities constitutes the most important threat to the wetland complex. This type of ecosystem is under extreme pressure from mining activities, with most of the páramos, as close as 25 km north of the type locality of *Pristimantis tiktik*, being already concessioned to mining companies (based on the information from the Mining Control and Regulation Agency of Ecuador—ARCOM). These activities consist in the extraction of large amounts of soil and rocks from the upper layers of the páramo, causing irreversible damage to this ecosystem. There is a high possibility that in the near future mining concession will be granted to the Oña, Nabón, Saraguro and Yacuambi wetland complex, since the legislative context offers no protection.

## Supporting information

S1 Appendix Additional specimens examined. (DOCX)

S1 File A 1-minute fragment of the advertisement call of *Pristimantis tiktik* sp.nov. (WAV)

S1 Table Primers used for PCR amplification. (DOCX)

S2 Table Voucher, GenBank accession numbers and localities for the specimens used in the phylogenetic analysis. (DOCX)

S3 Table Information regarding the call recordings and the bioacoustic measurements for each of the recorded males. Each of the recordings are from distinct males. Values are given as average ± SD (range) and *n* measured parameter.
(XLSX)

S4 Table Morphometric measurements of the *Pristimantis tiktik* sp. nov. type specimens. (XLSX)
