# Supplementary material for: A new minute Pristimantis (Amphibia: Anura: Strabomantidae) from the Andes of southern Ecuador
Source: PLoS One. 2018 Aug 29;13(8):e0202332. doi: 10.1371/journal.pone.0202332 (PMC6114709; doi:10.1371/journal.pone.0202332)
Supplement: S1 Table — (DOCX) [file pone.0202332.s003.docx]

A new minute *Pristimantis* (Amphibia: Anura: Strabomantidae) from the Andes of southern Ecuador

Paul Székely, Juan Sebastián Eguiguren, Diana Székely, Leonardo Ordóñez-Delgado, Diego Armijos-Ojeda, María Lorena Riofrío-Guamán, Dan Cogălniceanu

**S1 Table.** **List of primers used for PCR amplification of 12S rRNA, 16S rRNA, and RAG1 nDNA.**

| **Locus** | **Primer name** | **Direction** | **Sequence** | **Reference** |
| --- | --- | --- | --- | --- |
| *12S* rRNA | 12L29E | forward | AAAGCRTAGCACTGAAAATGCTAAGA | Heinicke MP, Duellman WE, Hedges SB. Major Caribbean and Central American frog faunas originated by ancient oceanic dispersal. Proceedings of the National Academy of Sciences. 2007;12;104(24):10092-7. |
|  | 12H10 | reverse | CACYTTCCRGTRCRYTTACCRTGTTACGACTT |  |
| *16S* rRNA | 16SC | forward | GTRGGCCTAAAAGCAGCCAC | Darst CR, Cannatella DC. Novel relationships among hyloid frogs inferred from 12S and 16S mitochondrial DNA sequences. Molecular phylogenetics and evolution. 2004;31(2):462-75. |
|  | 16SD | reverse | CTCCGGTCTGAACTCAGATCACGTAG |  |
| *RAG1* nDNA | R182 | forward | GCCATAACTGCTGGAGCATYAT | Cannatella, D. personal communication in Hedges SB, Duellman WE, Heinicke MP. New World direct-developing frogs (Anura: Terrarana): molecular phylogeny, classification, biogeography, and conservation. Zootaxa. 2008;1737: 1–182. |
|  | R270 | reverse | AGYAGATGTTGCCTGGGTCTTC |  |
