# Supplementary material for: A new minute Pristimantis (Amphibia: Anura: Strabomantidae) from the Andes of southern Ecuador
Source: PLoS One. 2018 Aug 29;13(8):e0202332. doi: 10.1371/journal.pone.0202332 (PMC6114709; doi:10.1371/journal.pone.0202332)
Supplement: S2 Table — (DOCX) [file pone.0202332.s004.docx]

A new minute *Pristimantis* (Amphibia: Anura: Strabomantidae) from the Andes of southern Ecuador

Paul Székely, Juan Sebastián Eguiguren, Diana Székely, Leonardo Ordóñez-Delgado, Diego Armijos-Ojeda, María Lorena Riofrío-Guamán, Dan Cogălniceanu

**S2 Table.** **Voucher, GenBank accession numbers and locality for the specimens used in the phylogenetic analysis.**

| **Species** | **Voucher number** | **GenBank accession no.** | | | **Locality** |
| --- | --- | --- | --- | --- | --- |
|  |  | ***12S*** | ***16S*** | ***RAG1*** |  |
| *Pristimantis andinognomus* | QCAZ45661 | - | KY967671 | KY967690 | Ecuador: Zamora Chinchipe, Reserva Tapichalaca |
| *Pristimantis andinognomus* | QCAZ45534 | - | KY967669 | KY967688 | Ecuador: Loja, Parque Nacional Podocarpus, guardianía Cajanuma |
| *Pristimantis bambu* | QCAZ46744 | - | KY967659 | KY967693 | Ecuador: Cañar, Reserva Mazar |
| *Pristimantis bambu* | QCAZ46708 | - | KY967673 | - | Ecuador: Cañar, Reserva Mazar |
| *Pristimantis ceuthospilus* | KU212216 | EF493520 | EF493520 | - | Peru: Cajamarca, Chota, 12 km W Llama |
| *Pristimantis chalceus* | KU177638 | EF493675 | EF493675 | - | Ecuador: Carchi, Maldonado |
| *Pristimantis cryophilius* | KU217863 | EF493672 | EF493672 | - | Ecuador: Azuay, 4 km W Laguna Torcadorn |
| *Pristimantis diadematus* | KU221999 | EU186668 | EU186668 | - | Peru: Loreto, Teniente Lopez |
| *Pristimantis galdi* | QCAZ32368 | EU186670 | EU186670 | EU186746 | Ecuador: Zamora Chinchipe, El Pangui |
| *Pristimantis imitatrix* | KU215476 | EF493824 | EF493667 | - | Peru: Madre de Dios, Cuzco Amazonico, 15 km E Puerto Maldonado |
| *Pristimantis mazar* | QCAZ27559 | - | KY967664 | KY967683 | Ecuador: Cañar, Reserva Mazar, La Libertad |
| *Pristimantis mazar* | QCAZ27572 | JF906315 | KY967666 | KY967685 | Ecuador: Cañar, Reserva Mazar, La Libertad |
| *Pristimantis melanogaster* | MHNSM56846 | EF493826 | EF493664 | - | Peru: Amazonas, N. Slobe Abra Barro Negro, 28 km SSW Leimebambe |
| *Pristimantis*  *muranunka* | MEPN14737 | - | KY967661 | KY967680 | Ecuador: Zamora Chinchipe, Cerro Plateado |
| *Pristimantis*  *muranunka* | MEPN14722 | - | KY967660 | KY967679 | Ecuador: Zamora Chinchipe, Cerro Plateado |
| *Pristimantis orestes* | KU218257 | EF493388 | EF493388 | - | Ecuador: Azuay, 7 km E Sigsig |
| *Pristimantis orestes* | QCAZ45464 | JF906323 | - | - | Ecuador: Loja, Parque Nacional Podocarpus, guardianía Cajanuma |
| *Pristimantis orestes* | QCAZ45646 | JF906324 | - | - | Ecuador: Loja, Parque Nacional Podocarpus, guardianía Cajanuma |
| *Pristimantis orestes* | QCAZ45556 | - | KY967670 | KY967689 | Ecuador: Loja, Parque Nacional Podocarpus, Lagunas del Compadre |
| *Pristimantis parvillus* | KU177821 | EF493352 | EF493352 | - | Ecuador: Pichincha |
| *Pristimantis phoxocephalus* | KU218025 | EF493349 | EF493349 | - | Ecuador: Chimborazo, 70 km W Riobamba via Pallatanga |
| *Pristimantis rhodoplichus* | KU219788 | EF493674 | EF493674 | - | Peru: Piura, Le Tambo |
| *Pristimantis simonbolivari* | QCAZ56567 | - | KY967676 | KY967695 | Ecuador: Bolívar, Bosque Protector Cashca Totoras |
| *Pristimantis simonbolivari* | KU218254 | EF493671 | EF493671 | - | Ecuador: Bolívar, Bosque Protector Cashca Totoras |
| *Pristimantis simonsii* | KU212350 | EU186665 | EU186665 | - | Peru: Cajamarca, S slope Abra Quilsh, 28 km NNW Cajamarca |
| *Pristimantis* sp. | QCAZ56535 | - | KY967675 | KY967694 | Ecuador: Azuay, Laguna Patococha |
| *Pristimantis* sp. | DHMECN3112 | - | KY967658 | KY967677 | Ecuador: Zamora Chinchipe, Reserva Tapichalaca |
| *Pristimantis spinosus* | KU218052 | EF493673 | EF493673 | - | Ecuador: Morona-Santiago, 10.6 km W Plan de Milogio |
| *Pristimantis tiktik* | MUTPL239 | MH668274 | MH668275 | MH708575 | Ecuador: Loja, 21 km E Urdaneta |
| *Pristimantis tiktik* | MUTPL247 | MH668161 | MH668276 | MH708576 | Ecuador: Loja, 14 km E Urdaneta |
| *Pristimantis unistrigatus* | KU218057 | EF493387 | EF493387 | EF493444 | Ecuador: Imbabura, 35 km E Pquela |
| *Pristimantis wiensi* | KU219796 | EF493377 | EF493668 | - | Peru: Piura: 12.7 km E Canchaque |
